# Barriers and facilitators of using health information technologies by women: a scoping review

**DOI:** 10.1186/s12911-023-02280-7

**Published:** 2023-09-05

**Authors:** Khadijeh Moulaei, Reza Moulaei, Kambiz Bahaadinbeigy

**Affiliations:** 1 Department of Health Information Technology, Faculty of Paramedical, Ilam University of Medical Sciences, Ilam, Iran; 2 School of medicine, Tehran University of Medical Sciences, Tehran, Iran; 3 Medical Informatics Research Center, Institute for Futures Studies in Health, Kerman University of Medical Sciences, Kerman, Iran

**Keywords:** Barriers, Facilitators, Health information technology, Women, Health

## Abstract

**Background and aim:**

Health information technologies play a vital role in addressing diverse health needs among women, offering a wide array of services tailored to their specific requirements. Despite the potential benefits, the widespread utilization of these technologies by women faces numerous barriers and challenges. These barriers can cause women to either reduce their usage of health technologies or refrain from using them altogether. Therefore, this review was done with the aim of identifying and classifying barriers and facilitators.

**Methods:**

Some databases, including PubMed, Web of Sciences, and Scopus were searched using related keywords. Then, according to the inclusion and exclusion criteria, the articles were evaluated and selected. Finally, the barriers and facilitators were identified and classified.

**Results:**

Out of 14,399 articles, finally 35 articles were included in the review. In general, 375 barriers (232 items) and facilitators (143 items) were extracted from the studies. After merging similar items, 121 barriers (51 items) and facilitators (70 items) identified were organized into five main themes (management, technological, legal and regulatory, personal, and data and information management). The most important barriers were “privacy, confidentiality, and security concerns” (n = 24), “deficiencies and limitations of infrastructure, software, hardware, and network” (n = 19), “sociocultural challenges” (n = 15), and “poor economic status” (n = 15). Moreover, the most important facilitators were “increasing awareness, skills and continuous education of women” (n = 17, in personal theme), “providing training services” (n = 14, in management theme), “simple, usable, and user-friendly design of technologies” (n = 14, in technological theme), and “providing financial or non-financial incentives (motivation) for women” (n = 14, in personal theme).

**Conclusion:**

This review showed that in order to use technologies, women face many barriers, either specific to women (such as gender inequality) or general (such as lack of technical skills). To overcome these barriers, policymakers, managers of organizations and medical centers, and designers of health systems can consider the facilitators identified in this review.

**Supplementary Information:**

The online version contains supplementary material available at 10.1186/s12911-023-02280-7.

## Background

Health Information Technology (HIT) is recognized as having the potential to empower women worldwide in economic, social, political and health domains [1]. In the field of health, technologies can provide potential for diagnosis, treatment, prognosis, and management of diseases, reducing travel and patient waiting time, reducing costs both for the health care system and for patients, more comfort and increasing the sense of self-efficacy [2]. Various studies [3–5] have also shown that technology can play an important role in improving women’s health. These technologies by improving women’s knowledge, attitudes, skills and lifestyle can make them easily accessible to care services, better manage diseases and increase the quality of care [2]. Pérez-Ferre et al. [6], reported a 65% reduction in the number of clinical visits for women with gestational diabetes mellitus (GDM) who used telemedicine. The primary benefits of doing this were improved work efficiency of health professionals (HPs) and better quality of life for women with GDM. Moreover, some studies [7–11] show that women extensively utilize health technologies during pregnancy to access crucial information, monitor their well-being, and address pregnancy-related concerns. Health apps, wearable devices, and telemedicine platforms have made it possible for pregnant individuals to track their prenatal progress, receive personalized health recommendations, and connect with healthcare professionals remotely [7–11]. Using these technologies, women stay informed about their pregnancy’s development, receive timely medical support, and actively engage in managing their health. This empowerment can lead to better maternal outcomes and enhanced prenatal care experiences [7–11].

However, there are always barriers to women’s use of technology around the world. The severity and scope of these problems for women are such that it may prevent them from accessing technology, using these technologies, and participating in jobs related to information and communication technology. Safiee et al.‘s systematic review [2] showed that barriers related to usability, technical problems, data privacy, and accuracy of reported data hinder the easy adoption of electronic technologies by pregnant women. Armstrong et al. [12], also pointed out that social and cultural views/biases held by society in general, lack of education and technical skills can also prevent women from using technologies. Literacy, education, language, place, time, economic status, skills and social cultural practices were among the other barriers identified in the study of Bertaux et al. [1]. Moreover, a survey has indicated a significant gender gap in Science, Technology, Engineering, and Mathematics (STEM) fields, resulting in women being underrepresented in ICT-related occupations worldwide [13]. Additionally, the gender digital divide persists, particularly in developing countries, where women have less access to information and communication technologies. According to the International Telecommunication Union (ITU), as of 2020, the global Internet user gender gap stood at 17%, with the disparity being more prominent in developing countries [14]. Furthermore, research has revealed the underrepresentation of women in tech leadership roles, while prevailing societal norms and stereotypes often discourage girls and women from pursuing careers in the tech industry [15]. To overcome these barriers, facilitators need to be identified and used in practice [2].

To our knowledge, no study has been done on barriers and facilitators of using health information technologies by women. Some review studies have focused on barriers to and facilitators of using eHealth in GDM [2], using information technology to educate women [1], and problems and opportunities of eHealth research [16]. So, the aim of this study was to review studies in the literature to identify and classify barriers and facilitators for health information technologies in relation to women’s health.

## Materials and methods

This scoping review was based on the Preferred Reporting Items for Systematic Reviews and Meta-Analyses (PRISMA) checklist [17].

### Definition

This study adopts the identical definition of health information technology (HIT) as the application of computer hardware and software that handles the storage, retrieval, sharing, and use of healthcare data and knowledge for communication and decision-making [18]. Nevertheless, this study focuses exclusively on HIT utilized by women and excludes those utilized by men. Specifically, the HIT solutions under examination are developed and intended exclusively for women and may address health issues that are unique to women.

In this review, any type of health information technology related to women, regardless of disease type, procedures, or health outcomes, was considered as health information technology. A barrier is defined as a condition, person, or thing that prevents the work, communication, or progress of other people, systems, or entities [19]. Conversely, a facilitator is defined as a person or something that helps another person, system, institution, or organization to accomplish tasks more efficiently, providing solutions to problems and making processes faster and easier [19]. In this scoping review, the scope, barriers and facilitators were considered regardless of their type.

### Information sources and search strategy

This scoping review was conducted on articles published until January 3, 2023. Four electronic databases were searched: PubMed, Web of Science, and Scopus. PubMed, Web of Science, and Scopus are chosen for health research due to their comprehensive coverage, multidisciplinary nature, and reliable indexing of scholarly literature [20, 21]. PubMed focuses on biomedical and life sciences research [20], Web of Science is known for rigorous journal selection and citation analysis [21], while Scopus offers broad coverage of scientific literature, including social sciences, making them valuable tools for interdisciplinary studies (National Center for Biotechnology Information, 2021; Clarivate Analytics, 2021; Elsevier, 2021) [22]. This combination provides researchers with diverse and reputable scholarly materials, forming a strong foundation for health-related studies [20–22]. Literature searches were performed using the following keywords and search strategy:

((Health informatics OR medical informatics OR technology OR health technology OR medical technology OR health information technology OR information technology OR biomedical technology OR digital technology OR eHealth) AND (women OR female) AND (challenge OR problem OR obstacle OR barriers) AND (opportunity OR facilitators OR adoption))

This search strategy was developed by RM, and KHM and finally approved by KB.

### Inclusion and exclusion criteria

Inclusion and exclusion criteria are presented below.

#### Inclusion criteria

- Articles published in the English language.
- Articles focused on health information technologies designed for women.
- Articles that discussed the proposed barriers or facilitators or both of them.

#### Exclusion criteria

- Articles focused on health information technologies designed for men.
- Articles focused on non-health technologies.
- Systematic reviews, review, and meta-analysis.
- Books.
- Book chapters.
- Letter to editor.
- Conference abstracts.
- Research protocol or protocol study.

### Study selection

**Fig. 1:**
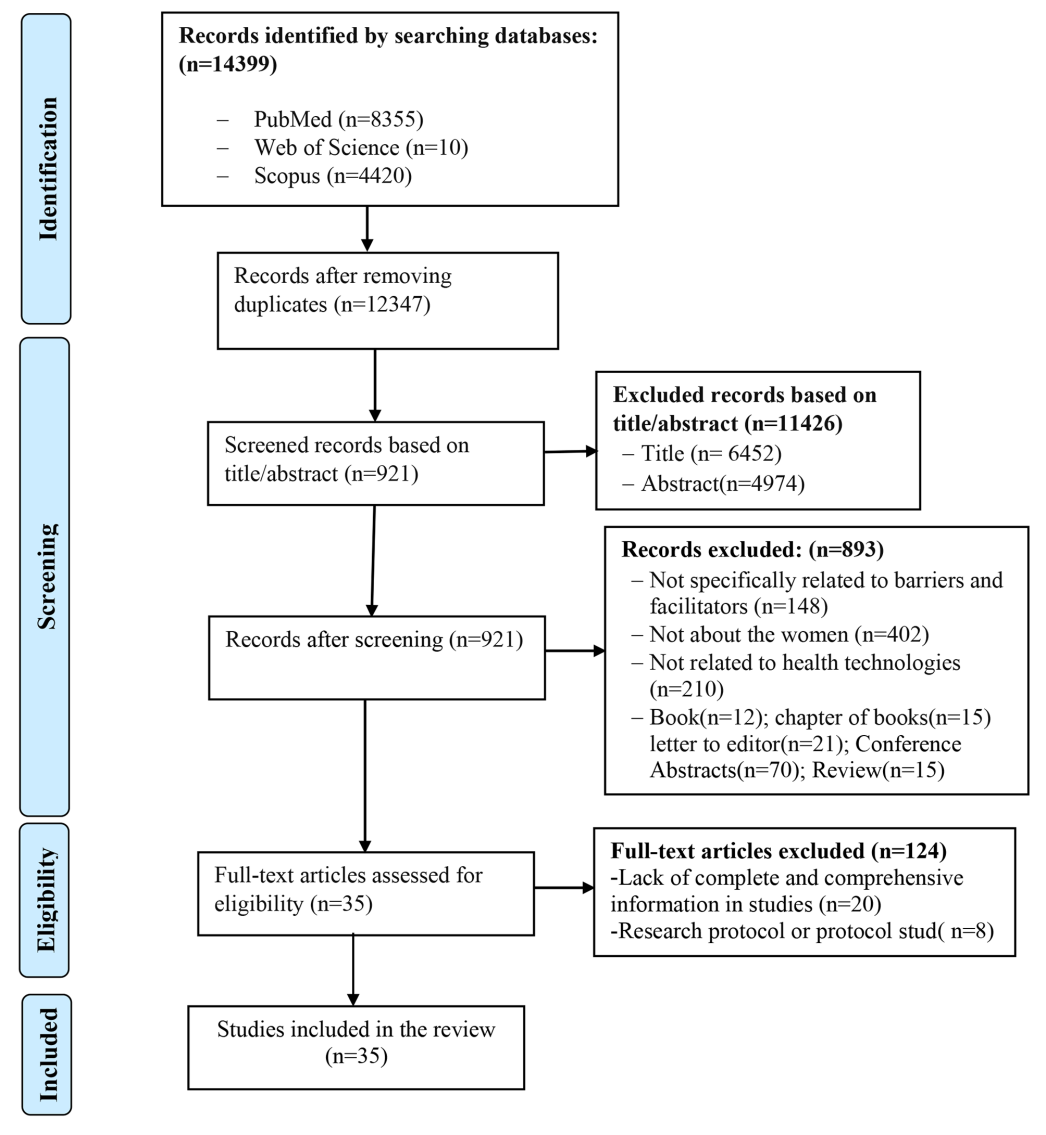
Selection of studies based on the PRISMA flowchart

After searching, the articles were imported into an EndNote library by KHM. After removing duplicate articles, the titles and abstracts were reviewed based on inclusion and exclusion criteria by all three authors. Then, the full text of the articles was reviewed (Fig. 1). For the articles for which we did not have access to the full text, we sent an email to the corresponding authors. In all stages of this section, both researchers (RM and KHM) independently reviewed the titles, abstracts and full text of the studies. In case of disagreement, the agreement was made by consensus between all three authors.

### Data extraction, charting, and synthesis

To collect the required data, we used a data extraction form and then extracted author(s), year of publication, country, study aim, type of women’s disease, barriers, facilitators, and other related results from the articles. After reviewing the articles, both researchers independently extracted data and information related to barriers and facilitators of health information technologies for women. If there was a difference between the results of these two researchers, these differences would be discussed in a joint meeting and finally an agreement would be reached.

Data analysis was done by content analysis method. The information obtained from each included article was discussed in different meetings. Finally, this information was organized in the form of main themes and sub-themes.

After the data were saved for further processing in MS Excel, one author (RM) checked all entered data (e.g., spell check, cell formatting) for data synthesis. Then descriptive statistics methods (frequency and percentage) were used to present the findings. The descriptive data obtained from the findings of the included studies were organized in the format of tables and figures based on the aims of this study (KHM and KB).

## Result

### Selection of the studies

In total, 14,399 articles were retrieved. After excluding duplicate articles, the remaining 12,347 articles were carefully reviewed and evaluated based on inclusion and exclusion criteria. Finally, 35 articles were included in the study. Figure 1 illustrates the search results and the study selection process.

### Description of the included studies

Details related to the description of included articles is provided in Appendix A.

### Distribution of studies by publication year, country, and women diseases

**Fig. 2:**
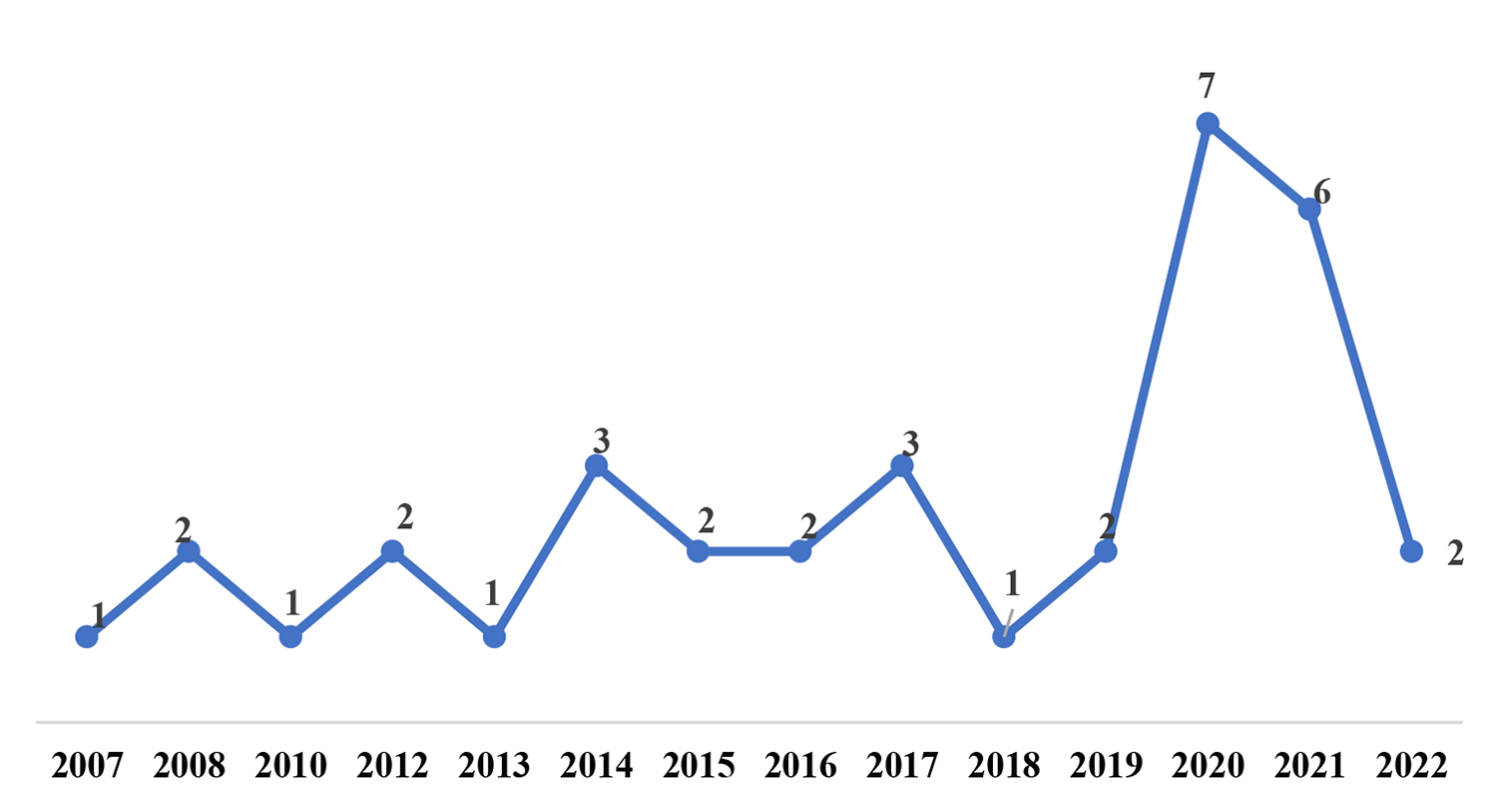
Distribution of studies by publication year

**Fig. 3:**
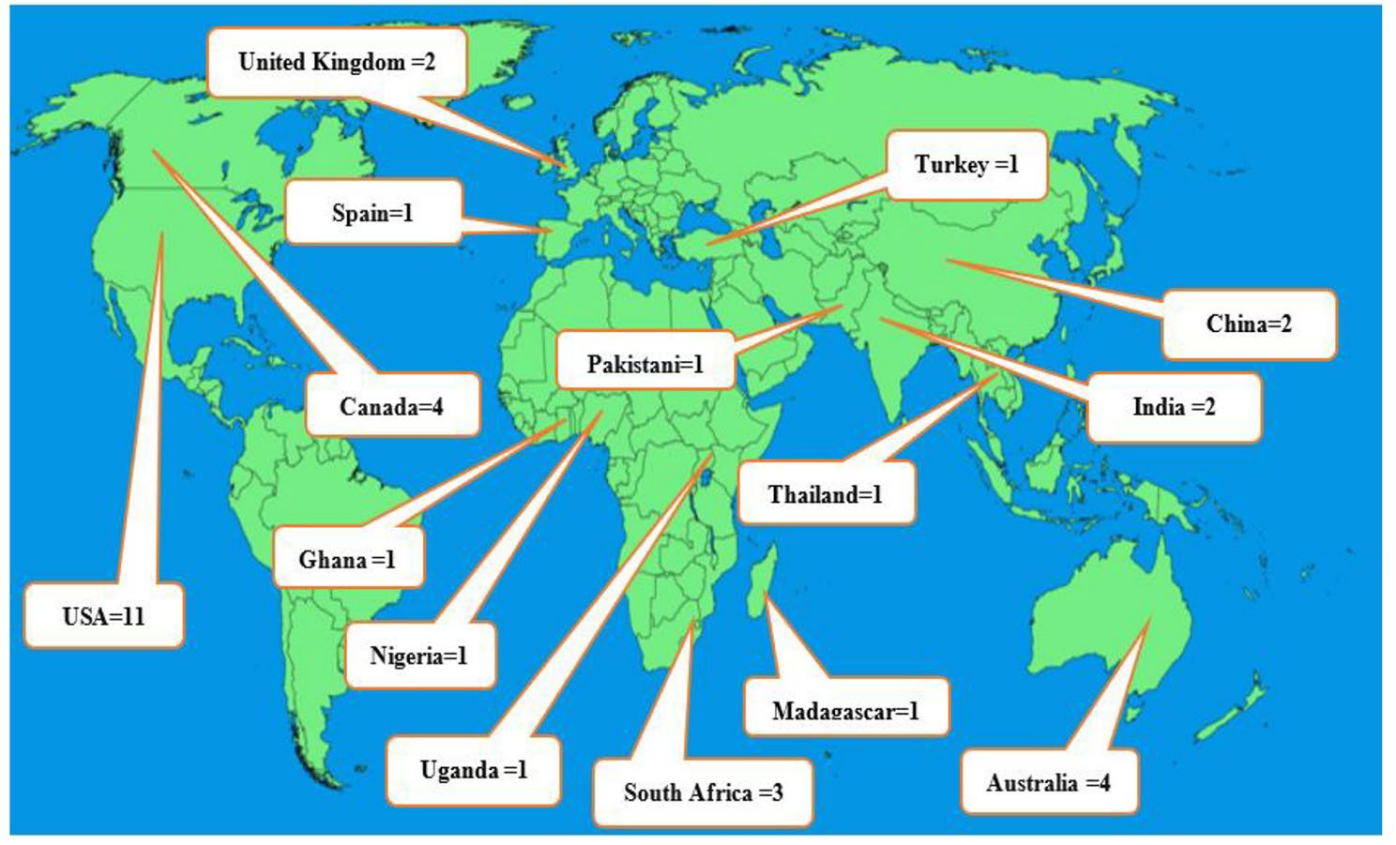
Included studies based on geographical location. *Note: The image of the map was taken from the Pxfuel website [44], we showed the frequency of included studies based on geographical locations on this image

Figure 2 shows that most studies were published in 2020 (n = 7, 20%) [23–29] and 2021 (n = 6, 17%) [30–35]. Moreover, most of the studies were conducted in the United States (n = 11, 31%) [26, 34–43] (Fig. 3).

**Fig. 4:**
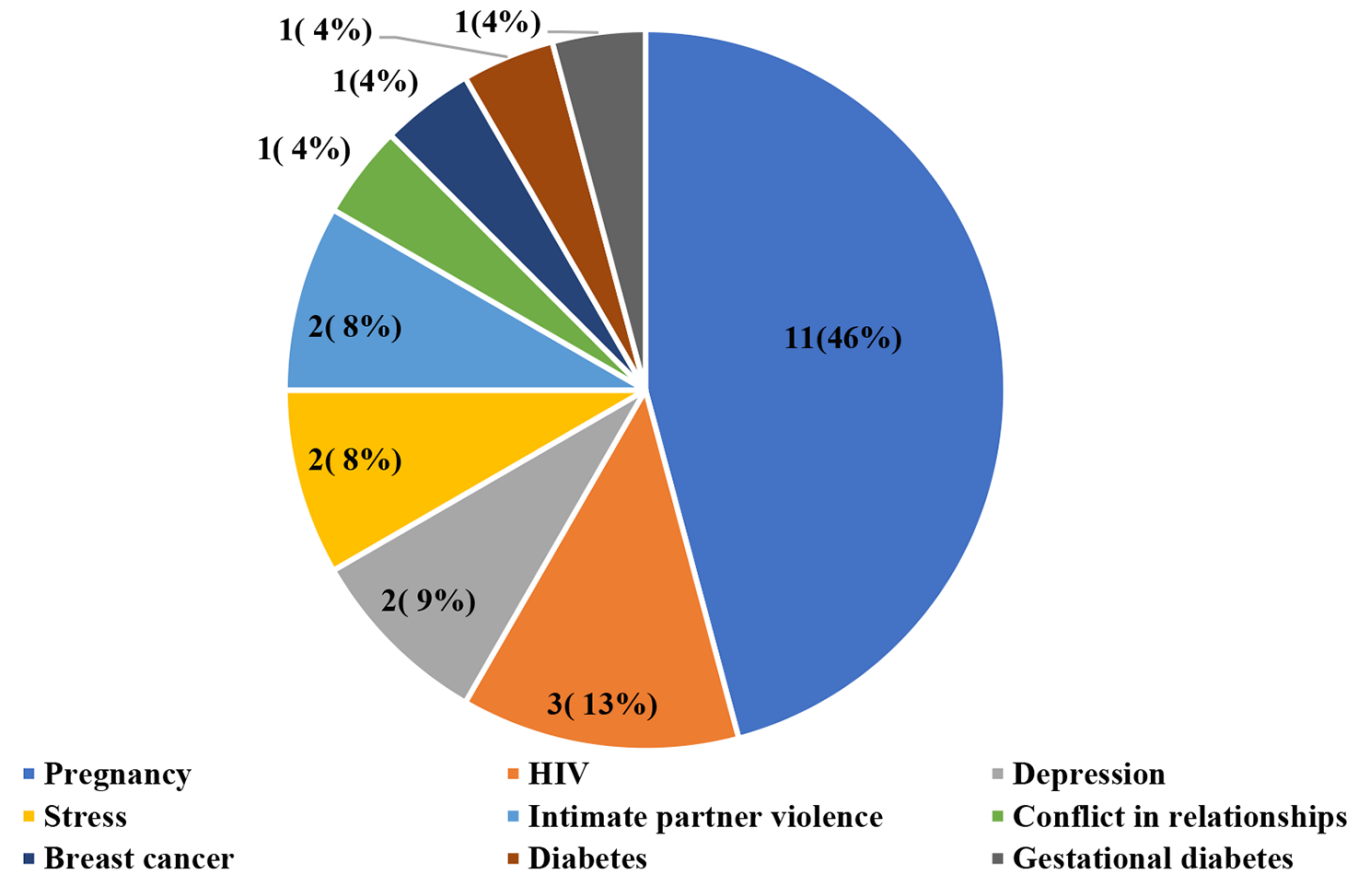
Distribution of studies on women’s diseases or other their conditions. *Note: It should be noted that in some studies, more than one disease, disorder or injurie were mentioned

The distribution of studies based on women’s diseases or other their conditions is presented in Fig. 4. Most digital technologies were used during pregnancy (n = 11, 46%) [24, 27, 29, 41, 45–51].

**Fig. 5:**
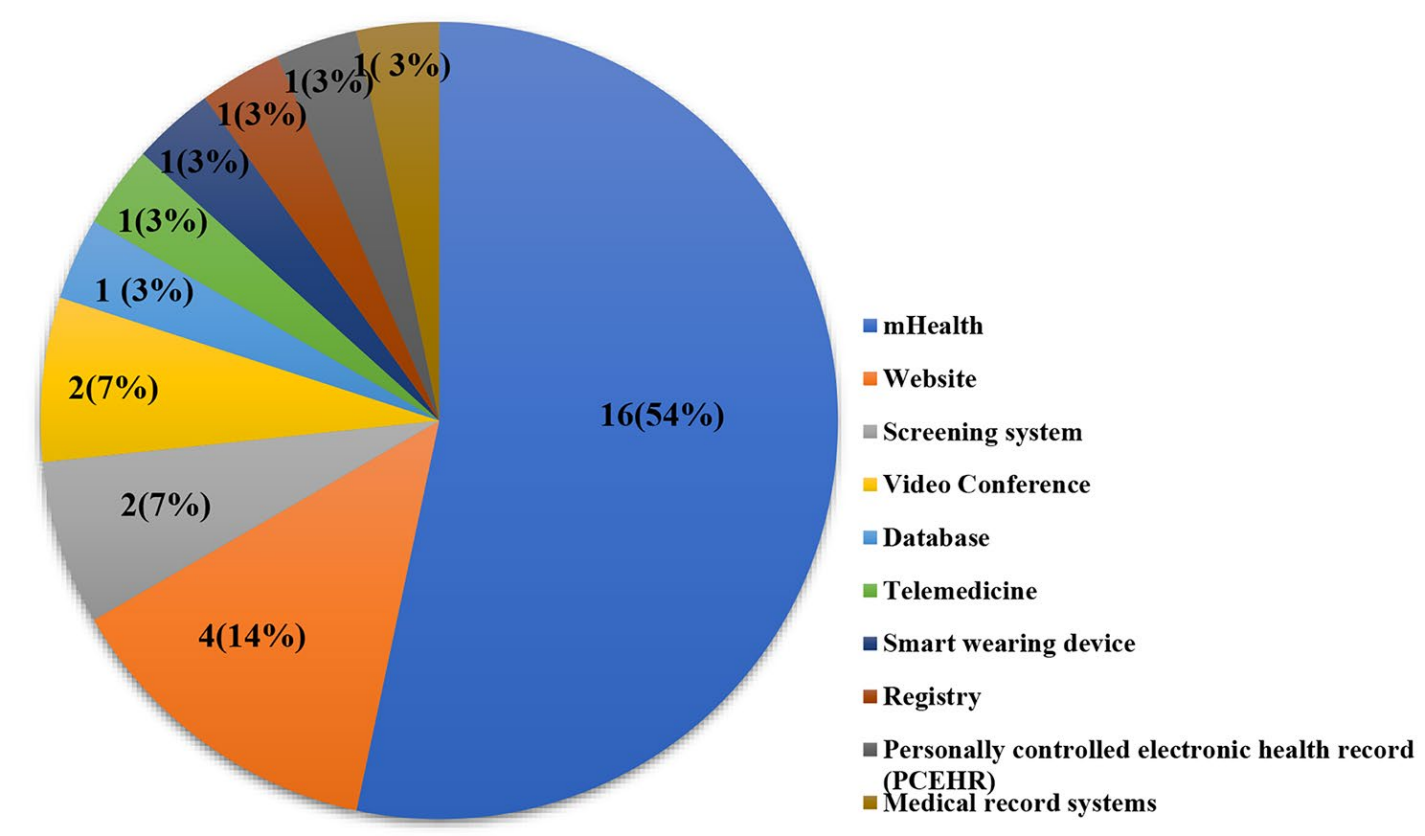
Types of digital interventions used to identify barriers and facilitators

According to Fig. 5, mHealth technologies were the most used health information technologies by women (n = 16, 64%) [23–26, 30, 35, 39–42, 45, 47–49, 52, 53]. Moreover, in some studies, in order to identify barriers and facilitators, women’s views on all health technologies were obtained. [27, 32, 34, 51, 54, 55].

### Barriers and facilitators of using health information technologies by women

In general, 375 barriers (232 items) and facilitators (143 items) were extracted from the included studies (more details in Appendix A). Finally, after merging similar items, 121 barriers (51 items) and facilitators (70 items) were identified. Then, we organized them into five main themes (management, technological, legal and regulatory, personal, and data and information management.). Barriers and facilitators related to management (15 barriers and 23 facilitators), personal (21 barriers and 14 facilitators), and technological (4 barriers and 24 facilitators) had the highest frequency. Tables 1 and 2 show barriers and facilitators of using health information technologies by women, respectively (more details in Appendix A).

Overall, based on frequency, “privacy, confidentiality, and security concerns“(n = 24) [23, 25–28, 31–36, 38–43, 48–53, 55], “deficiencies and limitations of infrastructure, software, hardware, and network” (n = 19) [25, 26, 29, 31, 32, 34, 36, 39, 40, 42, 43, 49–56], “sociocultural challenges” (n = 15) [23, 24, 29, 34, 35, 38, 40–42, 47, 53, 54, 56–58], and “poor economic status” (n = 15) [24–26, 32, 34, 38–40, 42, 47, 51, 52, 54, 56, 58] were the most important barriers (Table 1). Moreover, “increasing awareness, skills and continuous education of women“(n = 17) [25, 26, 29, 31, 34, 39–41, 43, 46, 49, 51–54, 57, 58], “providing training services” (n = 14) [26, 29, 34, 37–41, 46, 49, 51, 54, 57, 58], “simple, usable, and user-friendly design of technologies“ [23, 25, 28, 38, 41, 42, 45–49, 52, 54, 56] (n = 14), and “providing financial or non-financial incentives (motivation) for women” (n = 14) [23, 24, 26, 29, 33, 37, 40, 41, 46, 47, 49, 51, 54, 58] were the most important facilitators (Table 2).

Regarding management theme, “socio-Cultural challenges” (n = 15), and “sexual discrimination in technology” (n = 8) were the most identified barriers respectively. Also, in this theme “Providing training services"” (n = 14), “Reducing the anticipated stigma associated with disclosing abuse"” (n = 8), and “Financial support for women and through governments"” (n = 6) were the most common facilitators.

In the technological theme, the most identified barriers were “Deficiencies and limitations of infrastructure, software, hardware, and network” (n = 19), and “Poor design”, respectively (n = 9). Also, the most important facilitators of this theme were: “Simple, usable, and user-friendly design of technologies” (n = 14), and “Considering women’s preferences and needs in designing health information technologies” (n = 6).

In the legal and regulatory theme, the most important barrier was “Privacy, confidentiality, and security concerns” (n = 24). Among the five facilitators of this theme, the most important facilitator was “Enhancing privacy, confidentiality, security, and anonymity in unique ways“(n = 5).

In personal barriers theme, 22 barriers were identified. “Poor economic status“(n=(n = 14)), “limited access to various technologies“(n = 14), and “lack of technical skills and digital literacy” (n = 13) were the most important barriers, respectively. Moreover, 14 facilitators were identified in this theme. The most important of them were “increasing awareness, skills and continuous education of women“(n = 17), and “providing financial or non-financial incentives (motivation)” (n = 14) for women”.

In the data and information management theme, eight barriers and five facilitators were identified, respectively. These barriers and facilitators occurred with a frequency of one or two, based on the references.

It should be noted that among all the identified barriers, “Sexual discrimination in technology (for reasons such as less technological education of women than men, the need for permission to use technologies from husband/parents/partner, being under a conservative patriarchal regime, and others)” was the only barrier that exists exclusively for women. While other identified barriers can also exist in men’s society. The details of the barriers and facilitators for each theme are shown in Tables 1 and 2, respectively.

**Table 1:** Barriers of using health information technologies by women

Theme	Sub-them	Barriers (Ref)	Frequency based on references number	Total number of barriers identified for each theme
Management barriers	Lack of adequate training	Lacked technical support by companies or organizations [23, 37, 54]	3	13
		Lacked a training manual with technologies [37]	1	
		Lack of or inadequate knowledge and training for women about the health technologies [37]	1	
	Lack of insufficient resources	Lack of financial resources for development and maintenance [49, 54, 55]	3	
	Lack of planning, guidelines and protocols	Lack of long-term planning and estimation of the required resources [38]	1	
		Lack of precise and unique instruction and guideline for health technologies [37]	1	
	Environmental and sociocultural	Sociocultural challenges (such as institutional racism, discrimination by the healthcare system, and etc.) [23, 24, 29, 34, 35, 38, 40–42, 47, 53, 54, 56–58]	15	
		Sexual discrimination in technology (for reasons such as Less technological education of women than men, need permission to use technologies (from husband/ parents / partner), being under a conservative patriarchal regime, and etc.) [33, 34, 37, 46, 49, 53, 56, 58]	8	
		Inequality among women (including indigenous, those living in rural areas, ethnic and/or immigrant women, and women with partners other than men) [28, 29, 34]	3	
		Stigma surrounding the use of a digital mental health assessment [32]	1	
		Lack of social support [56]	1	
		Traditional beliefs [54]	1	
		Problems associated with bureaucracy [32]	1	
Technological barriers	Design, implementation and execution	Poor design (for example anonymous nature of some mobile phone services or poor usability) [26, 30–32, 41, 42, 45, 52, 53]	9	4
		Not using women’s individual preferences and values in designing health information technologies [33, 36]	2	
		Deficiencies and limitations of infrastructure, software, hardware, and network [25, 26, 29, 31, 32, 34, 36, 39, 40, 42, 43, 49–56]	19	
		Incompatibility of different systems [55]	1	
Legal and regulatory barriers	Violations, threats and concerns	Privacy, confidentiality, and security concerns [23, 25–28, 31–36, 38–43, 48–53, 55]	24	5
	Non considering ethical, legal, and regulatory principles	Lack of clear rules for establishing and using health technologies and protecting the privacy and security [29, 55]	2	
		Lack of legal authority to create a unique health identifier for individuals [55]	1	
		Absence of clear policies and legal rules to limit the release of collected data anonymous sharing of data [27, 55]	2	
		Lack of legal authority to collect and use data [55]		
Personal barriers	Inadequate training	Lack of literacy [24, 36, 37, 39, 43, 45, 46, 49, 51, 53, 58]	11	21
		Lack of training in use of health technologies [32, 51, 52, 54]	4	
		Lack of awareness of the benefits of technologies and services provided through them [53]	1	
		Lack of technical skills and digital literacy [31, 32, 34, 35, 38, 40, 43, 49–51, 54, 55, 57]	13	
	Low interest and motivation	Poor economic status [24–26, 32, 34, 38–40, 42, 47, 51, 52, 54, 56, 58]	15	
		Fear of inefficiency [32, 34, 38, 40, 41, 49, 52, 53]	8	
		Limited access to various technologies (for example not having mobile phones) [24, 25, 31, 34, 37, 38, 40, 41, 46, 49, 51, 53, 54, 57]	14	
		Language barriers [38, 39, 45, 51, 57, 58]	7	
		Reluctance to use technologies (For example, due to encountering hardware and software problems and not being able to solve them) [30, 32, 37, 40, 51, 53, 56]	7	
		Experienced personal anxiety or stress [28, 32, 37, 41, 52]	5	
		Existence of physical challenges such as left-handedness, poor eye-hand coordination with the mouse, and having to sit closer because of eyeglasses [37, 40]	2	
		Lack of self-confidence [34, 37, 53]	2	
		Not allocating enough time to use of health technologies [23, 26, 32, 40, 42, 51, 53]	7	
		Lack of experience or bad experience using technologies [41, 51, 53]	3	
		Fear that technologies such as a tablet could be broken, lost or stolen [39]	1	
		Not using body language [41]	1	
		Safety concerns [35]	1	
		High cost of services provided through technologies [53]	1	
		Shyness of women to provide information to the therapist [53]	1	
		Resistance to technology [32]	1	
		Difficult interaction with the health providers by technologies [55]	1	
Data and information management barriers	Inappropriate data collection methods	Using different methods to collect, disseminate and report data [55]	1	8
		Restrictions on data collection due to privacy concerns [45]	1	
	Poor data quality	Uncertainty about the timeliness and completeness of the recorded data [46]	1	
		Failure to update databases to ensure timely information [36, 41]	2	
		Concerns about the reliability and validity of unstructured data [55]	1	
		Bias in the interpretation of recorded data [55]	1	
	Lack of accurate and reliable information	Inaccurate or incomplete information shared through digital means [29, 30, 47]	3	
		Lack of trust in information provided through technologies [49]	1	

**Table 2:** Facilitators of using health information technologies by women

Theme	Sub-them	Facilitators (Ref)	Frequency based on references number	Total number of facilitators identified for each theme
Management facilitators	Providing adequate training	Providing training services [26, 29, 34, 37–41, 46, 49, 51, 54, 57, 58]	14	23
	Financing	Providing financial budget to support the spread of health technologies and internet services by governments [29, 38]	2	
		Providing relatively less expensive medical services [29]	1	
		Financial support for women and through governments [24, 29, 40, 46, 47, 58]	6	
	Managers’ performance	Establishing close collaboration between hospitals and professional institutes to improve the quality of online programs [29]	1	
		Administrative support for the use of the system [38]	1	
		Administrative support for personnel training [38]	1	
		Preventing possible spread of misinformation [30]		
	Environmental, and sociocultural	Reducing the anticipated stigma associated with disclosing abuse [25, 26, 28, 31–33, 41, 54]	8	
		Diminishing inequalities or disparities in health care [29, 42]	2	
		Social support [25, 51]	2	
		Face-to-face, interactive group sessions as most effective in communicating with culturally and linguistically diverse (CALD) communities [57]	1	
		Providing culturally tailored models of health care [57]	1	
		Creating spaces for communities, health workers and others to consider cultural attitudes to health communication and management [57]	1	
		Using different cultural patterns of knowledge acquisition [57]	1	
		Using the services of people from the same racial, cultural and linguistic background [57]	1	
		Cultural adaptation to perform appropriately with diverse populations [35]	1	
		Consideration of fasting or lifestyles/religious rituals [57]	1	
		Cultural consideration of the role of family in disseminating information (e.g., eldest son may do a majority of translating, or information sharing) [57]	1	
		Using different cultural patterns of knowledge acquisition [57]	1	
		Improved understanding of cultural [57]	1	
		Using an informal setting and opportunity for interaction to overcome culturally ingrained inhibitions [57]	1	
		Reducing gender-based barriers for women and girls in accessing healthcare services [58]	1	
Technological facilitators	Design, implementation and execution	Simple, usable, and user-friendly design of technologies [23, 25, 28, 38, 41, 42, 45–49, 52, 54, 56]	14	24
		Considering women’s preferences and needs in designing health information technologies [28, 32, 36, 40, 49, 56]	6	
		Designing technologies in a multilingual way [39, 42, 45]	3	
		Considering women’s computer literacy, IT knowledge, and technology experiences [30, 38]	2	
		Creating technology content in the form off and practical content [49, 54]	2	
		Promoting the use of the Internet and mobile electronic devices [29, 57]	2	
		Serving the systems both offline and online [39, 54]	2	
		Using of preferred community language(s) in designing technologies [57]	1	
		Greater flexibility in service delivery [57]	1	
		More use of voice instead of text in the design of technologies for literacy challenges [39]	1	
		Designing technologies according to age groups [40]	1	
		Improving convenience and information access [48]	1	
		System integration and managing the pre- and post-immunization experience [48]	1	
		Tailoring or personalizing technologies [28]	1	
		Designing systems in a scalable and expandable way to meet the needs of users with the progress of time and changing situations [38]	1	
		Providing computer kiosks in doctor’s waiting rooms to accessing the Internet anytime and anywhere [36]	1	
		Using cloud services as a feasible solution to reduce connectivity challenges [54]	1	
		Improving technical and practical solutions to improve implementation [23]	1	
		Constantly updating systems [46]	1	
		Developing technologies in an evidence-based manner [54]	1	
		Designing ICT technologies with a gender perspective [33]	1	
		Guarantee the widespread use of internet services by network operators [29]	1	
		Understand cultural factors influencing acceptability in technology design [35]	1	
		Creating informational content of technologies based on local needs and demands [56]	1	
Legal and regulatory facilitators	Fixing violations or threats	Enhancing privacy, confidentiality, security, and anonymity in unique ways [35, 36, 40, 50, 53]	5	5
	Appropriate setting of ethical, legal guidelines	Establishment of ground rules for use of technologies and protecting of women privacy [29, 43]	2	
		Providing the specific guidelines to govern social media use [51]	1	
		Considering specific regulations on confidentiality and access to data [50]	1	
		Developing strict legal regulations to protect data and avoid its exploitation for profit [27]	1	
Personal facilitators	Providing adequate training	Increasing awareness, skills and continuous education of women [25, 26, 29, 31, 34, 39–41, 43, 46, 49, 51–54, 57, 58]	17	14
		Providing education courses in basic and advanced computer training [37, 52, 53]	3	
		Planning and implementing training programs [37]	1	
		Use of bilingual educators to overcoming linguistic barriers [57]	1	
		Technical support (calling computer company for help and support, reading books and manuals, and etc.) [37]	1	
	Creating interest and motivation	Providing financial or non-financial incentives (motivation) for women [23, 24, 26, 29, 33, 37, 40, 41, 46, 47, 49, 51, 54, 58]	14	
		Introducing technologies and their benefits [41, 47]	2	
		Gain technical and computer skills [34, 40]	2	
		Using peer-led self-empowerment training programs [57]	1	
		Strengthening women consent processes [46]	1	
		Tailoring and personalizing the intervention [49]	1	
		Encourage learning about technology [37]	1	
		Ensuring the reliability of information provided through technologies [29]	1	
		Building trust in women towards technologies [29]	1	
Data and information management facilitators	Appropriate data collection methods	Importing clinical data from the EMR into the internet-based decision aids [36]	1	4
	Data quality assurance	Systematizing the process of gathering patient data [38]	1	
	Accurate and reliable information	Use multiple sources to verify information provided through technology [48]	1	
		Providing information in the form of evidence-based [49]	1	

## Discussion

This review showed that there are many barriers for women to use health information technologies, including management, technological, legal and regulatory, personal, and data and information management. Although these barriers may differ from one country to another, from one technology to another, and even based on the cultures of a region, removing some of these common barriers plays an important role in women’s use of these technologies. In the following, the most identified barriers and their facilitators are discussed.

### Management

Some barriers such as inadequate women training [23, 37, 54] or lacked technical support [37] are among the barriers that are often mentioned. The existence of these barriers can reduce or prevent the use of health technologies by women. Therefore, providing training services that can increase women’s technical knowledge and skills play an important role in women’s continuous use of technologies [26, 29, 34, 37–41, 46, 49, 51, 54, 57, 58]. Lack of insufficient resources such as lack of financial resources [49, 54, 55], lack of long-term planning and estimation of the required resources [38], and lack of precise and unique instruction and guideline for health technologies [37] are reported as other obstacles. These barriers can cause failures to develop, maintain, update, and implement a technology, or generally delay its continued progress. Other studies showed that having a program for secure sustainable funds should be considered from the beginning of the design and development phase of these technologies as one of the concerns of organizational managers [19].

Sociocultural [23, 24, 29, 34, 35, 38, 40–42, 47, 53, 54, 56–58], sexual discrimination in technology [33, 34, 37, 46, 49, 53, 56, 58], inequality among women [28, 29, 34], stigma [32], lack of social support [56], traditional beliefs and problems related to bureaucracy [32] were other barriers of the management theme. One of the important solutions in this field is planning to solve social cultural problems [35, 57, 58], diminishing inequalities or disparities [29, 42], and reducing the anticipated stigma [25, 26, 28, 31–33, 41, 54], and social support [25, 51].

### Technological

Deficiencies and limitations of infrastructure [26, 30–32, 41, 42, 45, 52, 53], poor design of technologies [26, 30–32, 41, 42, 45, 52, 53], not using women’s individual preferences and values in designing technologies [33, 36], and incompatibility of different systems [55] are among the barriers that can play an important role in women’s use and non-use of health technologies. According to various studies, promoting mobile electronic devices [29, 57], servicing technologies offline and online [39, 54], using cloud services [54], developing technologies in an evidence-based manner [54], and providing computer kiosks for Internet access [36] can be used to overcome infrastructural barriers.

Moreover, it is necessary to design technologies in a simple, usable, and user-friendly way, because a simple and user-friendly design can increase the continuous use of a technology by users [23, 25, 28, 38, 41, 42, 45–49, 52, 54, 56]. Also, using the preferences and views of women [28, 32, 36, 40, 49, 56] and a gender perspective [54] in designing a technology makes a technology designed according to the needs of women. This process can increase the motivation of women to use that technology [28, 32, 36, 40, 49, 56] and reduce or eliminate gender inequalities [54].

### Legal and regulatory factors

Improper formulation of principles and rules related to privacy, confidentiality and data security can create serious barriers for women to use health technologies. Many studies [23, 25–28, 31–36, 38–43, 48–53, 55] have already addressed women’s concerns about privacy and security in health technologies. Based on this, the lack of clear rules and proper planning to maintain privacy, security and confidentiality leads to the violation of women’s security and privacy, unauthorized access to their information and then misuse of this information [19]. Therefore, the development of strict legal regulations to maintain confidentiality, security, privacy, non-disclosure of information without permission and anonymity of women (for example, separating identity information from clinical data) are facilitators that can be useful in this field [27, 29, 35, 36, 40, 43, 50, 51, 53].

### Personal factors

Since the majority of users of any technology are women, considering personal factors and removing barriers are very important. Lack of technical skills and digital literacy [31, 32, 34, 35, 38, 40, 43, 49–51, 54, 55, 57], lack of training in the use of health technologies [32, 51, 52, 54], and lack of awareness of the benefits of technologies and services provided through them [53] are among the barriers to women’s use of health technologies. In order to overcome these problems, considering facilitators such as increasing women’s awareness and skills [25, 26, 29, 31, 34, 39–41, 43, 46, 49, 51–54, 57, 58], providing education courses in basic and advanced computer training [37, 52, 53], planning and implementing educational programs [37], using bilingual instructors to overcome language barriers [57], and technical support [37] are very useful.

In the personal theme, there were other barriers that reduced women’s interest and motivation to use technologies. Poor economic status [24–26, 32, 34, 38–40, 42, 47, 51, 52, 54, 56, 58], fear of inefficiency [32, 34, 38, 40, 41, 49, 52, 53], limited access to various technologies [24, 25, 31, 34, 37, 38, 40, 41, 46, 49, 51, 53, 54, 57], reluctance to use technologies [30, 32, 37, 40, 51, 53, 56], experienced personal anxiety or stress [28, 32, 37, 41, 52], lack of self-confidence [34, 37, 53], and resistance to technology [32] were among these barriers. Moreover, physical challenges (such as being left-handed, wearing glasses, etc.) [37, 40], lack of experience or bad experience using technologies [41, 51, 53], fear of breaking, losing or stealing technologies [39], not using body language [41], high cost of services provided through technologies [53], and difficult interaction with the health providers [55] were other barriers to reduce women’s motivation to use health technologies. Different studies pointed out that in order to increase the motivation of women, facilitators such as providing financial or non-financial incentives (motivation) [23, 24, 26, 29, 33, 37, 40, 41, 46, 47, 49, 51, 54, 58], introducing technologies and their benefits [41, 47], gaining technical and computer skills [34, 40], and using peer-led self-empowerment training programs [57] can be considered. Moreover, tailoring and personalizing the intervention [49], encourage learning [37], ensuring the reliability of information provided through technologies [29], and building trust in women towards technologies [29] can also increase the motivation of women to use technologies. However, it should be noted that the motivation of women in using technologies is a very important factor. If women do not have the necessary motivation to use a technology for any reason, they may never use that technology even if it is ideally designed.

### Data and information management

There are barriers that can affect the proper collection of data: using different methods to collect, disseminate and report data [55], and restrictions on data collection due to privacy concerns [45]. It seems that data collection in the form of importing clinical data from the EMR into the internet-based technologies such as decision aids can cause data collection in a suitable way [36]. In other words, importing data can eliminate the need for manual data entry and thus reduce the possibility of errors and mistakes in the data collection process [36]. Also, in importing data from EMR to other technologies, the possibility of violating the privacy and security of women’s data will be less, because data can be collected electronically, and it is easy to create restrictions on privacy and security [59].

Uncertainty about the timeliness and completeness of the recorded data [46], failure to update databases [36, 41], concerns about the reliability and validity of unstructured data [55], and bias in the interpretation of recorded data [55] are also among the barriers that can cause poor quality data. To overcome these barriers, systematizing the patient data collection process is very helpful [38]. Systematizing the data collection process can preserve privacy, reduce errors in manual data entry, and produce quality data [60].

Two other barriers in the theme of data and information management were inaccurate or incomplete information shared [29, 30, 47], and lack of trust in information provided through technologies [49]. Using multiple sources to verify information [48] and providing information in the form of evidence-based [49] were identified as facilitators for producing accurate and reliable information.

### Sexual discrimination in technology

According to the findings of this study, sexual discrimination in technology was the only barrier that exists exclusively for women, while other identified barriers can also exist in men’s society. One key factor contributing to sexual discrimination in technology is the disparity in technological education between men and women. Historically, there has been a gender gap in STEM (Science, Technology, Engineering, and Mathematics) fields, leading to fewer opportunities for women to gain adequate technological knowledge and skills [13]. This educational imbalance not only limits women’s entry into technology-related careers but also perpetuates the stereotypes that technology is a male-dominated domain. According to the opinion of Akinfaderin et al. [53], the requirement for women to seek permission from husbands, parents, or partners to use technologies is another significant aspect contributing to this barrier. This issue is deeply rooted in traditional societal norms, where women are expected to conform to the decisions and rules set by male figures in their lives. As a result, such restrictions hinder women’s autonomy and independence, making it difficult for them to fully explore and leverage technology for personal or professional growth.

Moreover, the existence of a conservative patriarchal regime further exacerbates sexual discrimination in technology. In societies with rigid gender roles and power structures, women often face systemic barriers that prevent them from participating in technological advancements and innovations. This cultural bias perpetuates the notion that women’s roles are confined to specific traditional domains, excluding them from pursuing opportunities in the technology sector [53, 61]. In societies with rigid gender roles and power structures, women often face systemic barriers that prevent them from participating in technological advancements and innovations. This cultural bias perpetuates the notion that women’s roles are confined to specific traditional domains, excluding them from pursuing opportunities in the technology sector [61].

The finding highlights the urgency of addressing gender disparities in the technology industry. To promote a more inclusive and diverse tech ecosystem, it is essential to develop targeted strategies that address the root causes of sexual discrimination in technology. Initiatives must focus on providing equal access to technological education and training for women, challenging and dismantling the norms that restrict women’s autonomy, and promoting policies that foster gender equality within the technology sector. Furthermore, raising awareness about these barriers is crucial in changing societal perceptions and dismantling stereotypes related to gender and technology. Encouraging open discussions, advocating for supportive policies, and providing mentorship and role models for aspiring female technologists can help dismantle the barriers that hinder women’s progress in this field.

Ultimately, fostering an environment that values and supports the contributions of women in technology will lead to more innovative and inclusive technological advancements. By recognizing and addressing the specific challenges faced by women, we can create a more equitable and diverse technology landscape that benefits society as a whole. This study’s findings serve as a catalyst for action, reminding us of the work that remains to be done to achieve true gender equality in the world of technology.

### Limitation of the study

This review has few limitations that can be considered as suggestions for future studies. First, studies from three databases (Scopus, PubMed, and Web of Science) published in English were only reviewed. Therefore, other studies published in other databases and non-English studies were not included. Second, we did not perform any kind of critical appraisal of individual evidence sources in this study. According to the PRISMA guidelines this is an optional section however this limitation can be considered in future studies.

## Conclusion

In this study, barriers and facilitators of using health information technologies by women were identified. A total of 51 unique barriers and 70 unique facilitators were identified and organized into five themes. Common barriers were “privacy, confidentiality, and security concerns”, “deficiencies and limitations of infrastructure, software, hardware, and network”, “sociocultural challenges”, and “poor economic status”. Moreover, common facilitators were “increasing awareness, skills, and continuous education of women” (in the personal theme), “providing training services” (in the management theme), “simple, usable, and user-friendly design of technologies” (in the technological theme), and “providing financial or non-financial incentives (motivation) for women” (in the personal theme).

In summary, this study sheds light on the factors that influence the use of health information technologies by women and provides actionable insights for stakeholders to improve the adoption and success of these technologies in the future. Considering these barriers and facilitators, managers, policymakers and designers of health systems can play a vital role to overcome the barriers to the use of health technologies by women. As an operational solution, managers and policy makers can use the results of this review to create a guideline or map to resolve socio-cultural barriers to increase the chances of women using health technologies.

### Electronic supplementary material

Below is the link to the electronic supplementary material.

Supplementary Material 1

## Data Availability

All data generated or analyzed during this study are included in this published article and its Additional files.

## Abbreviations

HIT: Health Information Technology, GDM:gestational diabetes mellitus
HPs: health professionals
