# Supplementary material for: Barriers and facilitators of using health information technologies by women: a scoping review
Source: BMC Med Inform Decis Mak. 2023 Sep 5;23:176. doi: 10.1186/s12911-023-02280-7 (PMC10478440; doi:10.1186/s12911-023-02280-7)
Supplement: Supplementary file 1 — Supplementary Material 1 [file 12911_2023_2280_MOESM1_ESM.docx]

**Barriers and facilitator of using health information technologies by women: a scoping review**

**Appendix A**. Full description and findings of included articles

| Facilitators | Challenges | Electronic intervention | Type of women's diseases or other their conditions | Purpose | Country | Journal | Author/ Year |
| --- | --- | --- | --- | --- | --- | --- | --- |
| - Considering the preferences and needs of women in therapeutic decision-making systems - Importing clinical data from the EMR into the internet-based decision aids - Providing computer kiosks in doctor's waiting rooms to overcome the challenges of accessing the Internet anytime and anywhere - Creating the necessary rules and measures to protect women' privacy | - Lack of access to high-speed Internet anytime and anywhere - Lack of literacy to use health technologies - Privacy concerns - Data security concerns - Not using patient's individual preferences and values in designing therapeutic decision-making systems - Not updating the data bases of decision aids that are not delivered over the Internet | Website | Menopausal | Identifying the challenges and opportunities of web-based decision support too in menopausal treatments on symptoms and risks | USA | Maturitas | Col/ 2007 [25] |
| - Encourage learning about technology - Providing education courses in basic and advanced computer training - Planning and implementing training programs - Technical support (calling computer company for help and support, reading books and manuals, and etc.) | - Lack of computer and digital literacy - Experienced personal anxiety or stress - Lack of self-confidence - Lacked a training manual with technologies Lacked technical support from the computer company - Lacked access to a health technology - Existence of physical challenges such as left-handedness, poor eye-hand coordination with the mouse, and having to sit closer because of eyeglasses - Reluctance to use technologies due to encountering hardware and software problems and not being able to solve them - Sexual discrimination in technology (Less technological education of women than men) | Website | Old age | Identifying the motivations and barriers to using a computer-based tool by elderly women | USA | Educational Gerontology | Rosentha/ 2008 [26] |
|  | - Concerns about information privacy - Difficult interaction with the physician by technologies - Lack of legal authority to create a unique health identifier for individuals - Absence of clear policies to limit the release of collected data and anonymous sharing of data - Increasing the number of mistakes with the computer-based technologies due to lack of technical skills - Lack of clear rules in establishing and using health technologies - Lack of legal authority to collect and use data - Ethical concerns for using EHR data - Incompatibility of different EHR systems - Using different methods to collect, disseminate and report data - Difficulty sharing patient data from EHRs to disease surveillance systems - Infrastructure constraints in support of EHR systems - Bias in the interpretation of recorded data - Lack of financial resources for development and maintenance - Poor data quality in EHRs - Problems related to reliability and validity of unstructured data | All digital health technologies | Smoking, stress, conflict in relationships, and safety | Evaluation of computer-based screening software for lifestyle risks, including smoking, stress, conflict in relationships, and safety, among female patients | Canada | Journal of medical Internet research | Ahmad /2008[43] |
| - Systematizing the process of gathering patient data - Educating patients to use technologies - Using telemedicine-based interventions in villages and hard-to-reach areas - Providing training services - Designing systems in a scalable and expandable way to meet the needs of users with the progress of time and changing situations - Considering women' computer literacy skills in designing systems - Considering women' attitudes towards IT - Considering the knowledge level of women about IT systems - Considering women's previous exposure to information technology systems - Administrative support for the use of the system - Providing financial budget to support the electronic devices - Administrative support for personnel training - Considering technological factors such as the user interface, ease of navigation, perceived usability, and etc. | - Privacy concerns - Fear of inefficiency - Poor economic status - Language barriers - Cultural barriers - Limited knowledge about health technology - Lack of access to IT-based interventions in rural or inner-city areas | Cancer screening system | Breast cancer | Collaborative development of an electronic reminder system for breast cancer screening and identifying its challenges and applications | USA | J Am Soc Inf Sci Technol | Lustria/ 2010 [27] |
| - Education of Vietnamese elderly women - Using training services - Creating spaces for communities, health workers and others to consider cultural attitudes to health communication and management - Using peer-led self-empowerment training programs - Providing culturally tailored models of health care - Face-to-face, interactive group sessions as most effective in communicating with culturally and linguistically diverse (CALD) communities - Using of preferred community language(s) - Use of bilingual educators to overcoming linguistic barriers - Using graphics and other visual to different levels of literacy - Using different cultural patterns of knowledge acquisition - Using the services of people from the same racial, cultural and linguistic background (as comprehension will cease to be an issue and establishing relationships of trust is easier) - Greater flexibility in service delivery - Improved understanding of cultural and lifestyle practices - Consideration of fasting or lifestyles/religious rituals - Cultural consideration of the family's role in disseminating information (e.g., the eldest son may do most of the translating or sharing of information) | - Lack of digital literacy and proficiency of women - Cultural issues - Language as the most significant barrier for communities in seeking information and accessing health services - Limited access to digital technology | Video Conferencing | Diabetes | Reporting the processes and results of a study on digital technology, diabetes and culturally and linguistically diverse (CALD) communities among women | Australia | Health Education Journal | O’Mara/ 2012 [45] |
| - Awareness raising of women - Addressing and protecting anonymity - Women's education | - Lack of internet and network access anywhere - High cost of services provided through technologies - Lack of literacy - Poor design (anonymous nature of some mobile phone services) - Sexual discrimination in technology (need permission to use phone (from parents / husband / partner)) - Low self-esteem/ self-confidence - Bad experience with the health services - Shyness of women to provide information to the therapist - Cultural challenges - Privacy and confidentiality issues - Fear of using mobile phone services - Not having enough time to use mobile phone services - Patients' fear of information disclosure - Low awareness of health services provided through mobile technologies - Lack of interest in using health technologies - Lack of sufficient training to use the services provided through mobile phones - Logistical or practical issues such as poor cell phone network, not having a phone, or phone battery not charging - Lack of trust (of authenticity of service) - Infrastructure issues such as poor network quality and inadequate power supply | mHealth | Sexual and reproductive health (SRH) | Identifying the opportunities and limitations of using new media and mobile phones in the access of Nigerian women and girls to sexual and reproductive health information and services. | Nigeria | Afr J Reprod Health | Akinfaderin / 2012[41] |
| - Providing adequate training - Simple and user-friendly design of technologies | - Privacy and confidentiality concerns - Poor usability - Inadequate training - Feelings of anxiety and fear about the multimedia capabilities of the phone - Poor socioeconomic status - Hardware and/or software challenges | mHealth | HIV-Positive Pregnant Women | Describing the acceptability and feasibility of mobile phones as a tool for data collection among pregnant women with H | South Africa | J Med Internet Res | van Heerden/ 2013 [40] |
| - Providing financial or non-financial incentives (motivation) for women - Providing the specific guidelines to govern social media use - Women’s education and providing training services - Social support | - Lack of digital literacy to use health technologies - Lack of training in use of health technologies - Confidentiality and privacy issues - Motivational concerns due to the high perception of risk associated with social media - Lack of availability of affordable ICT infrastructure and resources - Lack of the technical skills required to use ICT - Lack of opportunities for ICT use - Poor socioeconomic status - Culturally concerns - Linguistically concerns - lack of skills regarding the use of health technologies - lack of experience regarding the use of health technologies | Information and communication technologies (ICTs) | Pregnancy | Identifying midwives' attitudes and experiences of using information and communication technology to identify effective factors in improving prenatal care | Australia | Women Birth | Dalton/2014 [39] |
| - User-friendliness of information and communication technologies (ICT) - Creating informational content of technologies based on local needs and demands - Understand the complexity and multidimensionality of the information needs of women | - Lack of digital skill and literacy - Being under a conservative patriarchal regime - Sexual discrimination in technology (the possession and independent use of information and communication technology (ICT) equipment (especially mobile phones, computers) are still considered to be a “man’s job” and women are incapable) - Lack of enthusiasm and willingness of women to operate health technologies - Barriers of infrastructural underdevelopment - Lack economic resources to using innovative initiatives - Lack of social support - Cultural norms of gender segregation - Disruption in internet connectivity | Database | - | Establish an Information and Communication Center (ICC) to improve women's health information | Pakistani | BMC Women's Health | Zakar/ 2014 [44] |
| - Addressing privacy, and control concerns - Considering specific regulations on confidentiality and access to data | - High speed internet access challenges - Personal familiarity with computers - Access to high-speed computer equipment - Confidentiality and privacy issues | Personally controlled electronic health record (PCEHR) and medical record systems: | Pregnancy | Identifying medical record preferences according to the opinion of pregnant women and barriers to adoption of the personally controlled electronic health record (PCEHR) in their opinion | Australia | Telemed J E Health | Quinlivan/2014 [38] |
| - Providing training processes to women - Creating information provided through technologies in the form of evidence-based and practical content - Optimizing user engagement and experience - Usability of mobile apps - Maximizing engagement and motivation of women in tailoring and personalizing the intervention - Needing to continually updated and evidence-based information to earn trust of women - Designing technologies to suit women’s needs | - Lack of trust in information provided through mobile technologies - Privacy breaches concerns - Lack of health funds for technologies - Lack of familiarity with technology - Fear of loss of information control through technologies - Uncertainty as to who should be responsible for ensuring high-quality mHealth - lack of information technology know-how - Women's lack of access to the Internet - Women's lack of access to the health technologies such as mobile phones - Gender inequality for access to technologies | mHealth | Pregnancy | Investigating opinions of women and health professionals about mHealth to help women eat well, be physically active and gain appropriate amounts of weight during pregnancy. | Australia |  | Willcox/ 2015 [37] |
| - Multilingual technology design - More use of voice instead of text in the design of technologies for literacy challenges - Providing training services - Providing services offline when facing network connectivity issues | - Literacy issues - Language challenges - Concerns about privacy - Internet access problems - Fear that technologies such as a tablet could be broken, lost or stolen - Financial issues | mHealth | Depression | Determining the views and attitudes of clinic staff towards the use of mHealth to perform perinatal depression screening | USA | Family practice | Pineros-Leano/ 2015 [28] |
| - Addressing security and privacy concerns - Gain technical and computer skills - Eliminating financial worries by governments - Considering financial incentives - Designing technologies according to age groups - Training in e-health literacy and using health technologies - Educating women to increase their motivation | - Concerns about the security and privacy - No computer or smartphone - Sociocultural challenges (such as institutional racism, discrimination by the healthcare system, and etc.) - No reliable Internet accesses - Not enough time to use technologies - Need to technical/computer skills - Financial constraints - Low motivation - Fear and mistrust of the healthcare system - Challenges caused by people's age (such as visual impairment) | mHealth | - | Identifying reasons for African American women's willingness and unwillingness to participate in eHealth/mHealth research | USA | Telemed J E Health | James/ 2016 [29] |
| - Reducing the anticipated stigma associated with disclosing abuse - Introducing technologies and their benefits to users - Providing financial or non-financial incentives (motivation) for women - Designing technologies in a simple and user-friendly way - Educating women | - Concerns about security, confidentiality, and privacy - Lack of trust in technologies - Fear and anxiety about the use of technologies - Not using body language, eye contact, tone of voice, and other gestures in communicating with service providers - Lack of experience with technology - Usability challenges - No internet accesses - Failure to update technologies and their information content - Cultural beliefs - Limited access to various technologies (for example not having mobile phones) | mHealth | Intimate partner violence (IPV) during pregnancy | Identifying the views and opinions of women who experience intimate partner violence in the use of mHealth technology | USA | J Med Internet Res | Bacchus/2016 [30] |
| - Improving convenience and information access - Ease of use associated using a mobile app - Using multiple sources to verify providing information by technologies - System integration and managing the pre- and post-immunization experience | - Concerns regarding the privacy and security of personal health information | mHealth | Pregnancy | Identifying barriers and facilitators of mobile application adoption | Canada | [J Public Health (Oxf)](https://www.ncbi.nlm.nih.gov/pmc/articles/PMC5939700/) | Burgess/ 2017 [36] |
| - Educating women - Developing technologies in an evidence-based manner - Serving the systems both offline and online - Using cloud services as a feasible solution to reduce connectivity challenges. | - Lack of technical support - Infrastructure problems such as limited access to electricity, and lack of telephone lines and etc. - Medical and IT equipment limited in rural and remote areas - Lack of strong internet network - Low IT literacy - lack of funding for ICT infrastructure - Low bandwidth in rural areas - Financial dependence of women - Traditional beliefs - Lack of training - Cultural concerns | All digital health technologies | - | Investigating the feasibility of e-health solutions to reduce delays in maternal health care in remote areas | Ghana | BMC Med Inform Decis Mak | Pagalday-Olivares/2017 [42] |
| - Reducing gender-based barriers for women and girls in accessing healthcare services - Providing financial or non-financial incentives (motivation) for women Education - Financial support through governments - Planning and providing training services | - Lack of literacy - Gender-based barriers for women and girls in accessing healthcare services - Financial barriers to access healthcare services - Socio-cultural barriers to access healthcare services - language concerns | Telemedicine |  | Investigating the role of telemedicine in reducing gender-based barriers for women and girls in rural Nepal to access health services | Thailand | Telemat Inform | Parajuli/2017 [46] |
| - Addressing health care disparities - User-friendliness and easy use of technologies - Multilingual technologies - Conduct usability evaluation before technology release | - Poor usability - Privacy concern with women’s health apps - Technical issues - Bad economic status - Sociocultural status - Time constraints | mHealth | - | Evaluating the usability and acceptability of Healthier Women, a mobile-based application to enhance informed health choices | USA | J Obstet Gynecol Neonatal Nurs | Reyes/ 2018 [31] |
| - Establishment of ground rules for use of technologies - Increasing technological literacy | - Anonymity and privacy concerns - Low speed internet connection - Low literacy levels - Technical challenges | Video conferencing system | HIV | Feasibility evaluation of an online video conferencing system to further adapt computer-mediated communication (CMC) to facilitate simultaneous focus group discussions among transgender women. | USA | J Med Internet Res | Wirtz/2019 [32] |
| - Intuitive technical ease of use - Introducing technologies and their benefits - Reducing financial barriers among women | - Financial obstacles - Inaccurate or incomplete information shared through digital means - Cultural aspects | mHealth | Pregnancy | Identifying and determining the challenges and opportunities of mobile health for health care during pregnancy | Madagascar | JMIR Mhealth Uhealth | Muller/2019 [35] |
| - Use of financial and non- financial incentives - Ease of using the mobile phone - Improving technical and practical solutions to improve implementation | - Not allocate enough time to use of health technologies due to busy schedules - Lack of technical support - Privacy and confidentiality issues | mHealth |  | Identification of barriers and facilitators of mobile phone use to completing daily by women with HIV | South Africa | PLoS ONE | Dietrich/ 2020 [12] |
| - Supporting women with microenterprise interventions - Supporting women with financial incentives such as economic empowerment programs like non-refundable cash | - Lack of access to technologies - Lack of literacy - Poverty of women - Cultural differences | mHealth | Pregnancy | Identifying maternal health barriers among illiterate pregnant women in rural Uganda and highlighting the potential of mobile health technologies | Uganda | J Family Med Prim Care | Tumuhimbise/2020 [13] |
| - Users’ convenience: perceptions that these advanced technologies would offer convenience - Social support - Awareness raising of women Supporting the security and privacy - Reducing anticipated stigma by sex workers women | - Lack of access to technologies - Privacy issues - Potential threats to security - Financial instability - Internet access | mHealth | HIV | Identify potential facilitators and barriers to incorporating mobile phones and advanced technologies (such as biometric identification methods, mobile apps, and chatbots) to deliver HIV-related interventions to women sex workers (FSW) living with HIV | South Africa | Mhealth | You/ 2020 [14] |
| - Women education and planning and providing training services - Intense personal encouragement to persuade them to use the app | - No privacy to do the meditation - Poor usability - Technical problems with installation or use - Lack of access to a smartphone or computer - Not enough time - Lack of literacy | mHealth | Stress | Assessing the feasibility and acceptability of a mobile phone-based mindfulness program in stressed women | USA | JMIR Mhealth Uhealth | Rung/ 2020 [15] |
| - Developing strict legal regulations to protect data and avoid its exploitation for profit | - Security and privacy breach concerns - Absence of strict legal regulations for data protection | All digital health technologies | Pregnancy | Identifying and quantifying the ethical challenges of digital health technologies among Indian pregnant women | India | Bull World Health Organ | Gopichandran/ 2020 [16] |
| - Removing societal and cultural stigma leading to feelings of shame and guilt - Tailoring or personalizing technologies for increasing the motivation - Needing to further explore the particular needs and experiences of women - Attractiveness and user-friendliness of technologies | - Security and privacy breach concerns - Feeling anxious or upset - Inequality among women who face the most fundamental barriers to support (including indigenous, ethnic and/or immigrant women, those living in rural areas, and women with partners other than men) | Website | Intimate partner violence (IPV) | Examining the effects of an online health intervention for women experiencing intimate partner violence | Canada | BMC Public Health | Ford-Gilboe/ 2020 [17] |
| - Providing relatively less expensive medical services - Building trust in women towards technologies - Diminishing inequalities in health care due to its convenience and cost-effectiveness, especially in developing countries or regions - Improving women's awareness through providing various educational services - Establishing close collaboration between hospitals and professional institutes to improve the quality of online programs - Ensuring the reliability of information provided through technologies - Promoting the use of the Internet and mobile electronic devices - Guarantee the widespread use of internet services during the disease's outbreak by network operators - Financial support for women and through governments - Set the relevant rules to regulate and protect the privacy of pregnant women when using online antenatal care services - Providing training services | - Concerns about the reliability of online gestational information - Lack of access to internet and strong electronic devices by women - Absence of clear laws to regulate and protect the privacy - Inequality among women | Website | Pregnancy | Identifying opportunities and challenges of online prenatal care during the COVID-19 pandemic | China | J Med Internet Res | Wu/ 2020 [18] |
| - Understanding women's experiences of mHealth to ensuring acceptance and use - Providing accurate and reliable information - Preventing possible spread of misinformation. | - Poor usability - Reluctance to use technologies - Concerns about accuracy and misinformation shared online | mHealth | Gestational diabetes | Examining the opinions and experiences of women with previous gestational diabetes, regarding the use of mHealth resources before, during and after pregnancy | United Kingdom | Midwifery | Edwards/ 2021 [19] |
| - Increased awareness and understanding of technologies benefits by training - Reducing Stigma | - Usability issue - Data protection barriers - Poor technical skills - Poor access to technology and/or the internet in rural or deprived areas - Privacy and confidentiality concerns | Screening system |  | Development and evaluation of a digital tool for perinatal mental health | United Kingdom | J Med Internet Res | Martin-Key/ 2021 [20] |
| - Removing societal and cultural stigma leading to feelings of shame and guilt - Meeting women’ needs and preferences by technologist | - Financial constraints for access to health services - Privacy and security problems - Poor usability - Poor technical skills - Stigma surrounding the use of a digital mental health assessment - Problems associated with bureaucracy - Infrastructure concerns - Distress and a general fear of technology - Skepticism regarding the credibility of the tool - Beliefs associated with a digital tool being inappropriate to diagnose mental health conditions - Reluctance to using the IT interventions - Resistant to change - Lack of training - Time constraints | All digital health technologies | Postpartum Depression | Using a web-based platform to provide skills and psychosocial education to help manage postpartum depression in Colombian women. | Canada | J Med Internet Res | Lackie/ 2021 [21] |
| - Designing ICT technologies with a gender perspective - Removing societal and cultural stigma leading to feelings of shame and guilt | - Lack of pay attention to the woman needs in the designing of a technology due to the inherent patriarchal component of the current technology - Restrictions on data collection due to privacy concerns - Sexual discrimination in technology | Wearing device | Intimate partner violence | Identifying the challenges and opportunities of new technology proposals to deal with intimate partner violence | Spain | Technology in Society | Gorfinkiel, 2021 [22] |
| - Use of educational programs and training services - Advancing women technical skills | - Limited access to the internet - Lack of digital literacy and skills - Limited technology access in correctional facilities - Concerns regarding surveillance and privacy - Financial challenges - Social challenges - Unequal computer access for women of differing races - Lack of confidence for using ICTs (digital efficacy) - Sexual discrimination in technology - Fear of inefficiency - Social inequality | Internet and all digital health technologies | Marginalized women during COVID-19 | Identifying digital divides in marginalized women during COVID-19 | USA | Information, Communication & Society | Blomberg/ 2021 [23] |
| - Cultural adaptation to perform appropriately with diverse populations - Understand cultural factors influencing acceptability in technology design - Privacy and confidentiality assurance | - Privacy and confidentiality concerns - Cultural factors influencing acceptability - Technical difficulties such as phones not connecting to the charger properly, or environmental factors such as protecting the device from rain - Safety concerns created by the use of technologies | mHealth | Mental disorders | Assessing the feasibility and acceptability of mobile phone devices to improve maternal mental health services in low-resource environments | USA | BMC Med Inform Decis Mak | Maharjan/ 2021 [24] |
| - Financial support from government programs - Sufficient training - Strengthening women consent processes - Constantly updating systems - سDesigning technologies in an intuitive and user-friendly way | - Lack of literacy - Sexual discrimination in technology - Uncertainty about the timeliness and completeness of the recorded data - Lack of access to technologies (for example, not having personal mobile phones) | Registry | Pregnancy and postpartum | Understanding the challenges and opportunities effective in maintaining accurate, complete and timely digital records of pregnant and postpartum women in the Indian public health system. | India | BMJ Open | Scott /2022 [34] |
| - Strengthening the usability - Considering the capability of multi-lingual modes (Arabic, Turkish, and English) in the design of technologies | - Restrictions on data collection due to privacy concerns - Failure to provide clear instructions to the user to use the tool - language (the language of the digital tool designed was not the same as the language of women) - Lack of access to high-speed and wireless internet | mHealth | Pregnancy | Investigating the barriers and challenges of Syrian refugee women's health care in Turkey and their perception of a maternal and child health mobile application | Turkey | Frontiers in public health | Meyer/2022 [33] |
